# Associations of Residential Brownness and Greenness with Fasting Glucose in Young Healthy Adults Living in the Desert

**DOI:** 10.3390/ijerph18020520

**Published:** 2021-01-10

**Authors:** Hector A. Olvera-Alvarez, Matthew H. E. M. Browning, Andreas M. Neophytou, Gregory N. Bratman

**Affiliations:** 1 School of Nursing, Oregon Health and Science University, 3455 SW U.S. Veterans Hospital Rd., Portland, OR 97239, USA; 2 Virtual Reality and Nature Lab, College of Behavioral, Social and Health Sciences, Clemson University, Clemson, SC 29634, USA; mhb2@clemson.edu; 3 Department of Environmental and Radiological Health Sciences, Colorado State University, Fort Collins, CO 80523, USA; andreas.neophytou@colostate.edu; 4 School of Environmental and Forest Sciences, University of Washington, Seattle, WA 98195, USA; bratman@uw.edu

**Keywords:** green space, arid, diabetes, Hispanics, built environment, biophilia

## Abstract

Evolutionary psychology theories propose that contact with green, natural environments may benefit physical health, but little comparable evidence exists for brown, natural environments, such as the desert. In this study, we examined the association between “brownness” and “greenness” with fasting glucose among young residents of El Paso, Texas. We defined brownness as the surface not covered by vegetation or impervious land within Euclidian buffers around participants’ homes. Fasting glucose along with demographic and behavioral data were obtained from the Nurse Engagement and Wellness Study (*n* = 517). We found that residential proximity to brownness was not associated with fasting glucose when modeled independently. In contrast, we found that residential greenness was associated with decreased levels of fasting glucose, despite the relatively low levels of greenness within the predominantly desert environment of El Paso. A difference between the top and bottom greenness exposure quartiles within a 250 m buffer was associated with a 3.5 mg/dL decrease in fasting glucose levels (95% confidence interval: −6.2, −0.8). Our results suggest that within the understudied context of the desert, green vegetation may be health promoting to a degree that is similar to other, non-desert locations in the world that have higher baselines levels of green.

## 1. Introduction

Despite an unprecedented rate of global urbanization in recent decades, human evolution has primarily occurred in natural environments [1,2,3,4]. A mounting body of evidence shows that exposure to elements of these natural environments (e.g., trees, vegetation, lakes, and oceans) contributes to human health and well-being [5,6,7,8]. For example, exposure to greenness (i.e., trees, vegetation) has been associated with better sleep [9,10,11,12,13], cognitive function [14,15,16], and immune function [17,18,19], as well as reduced stress [15,20,21,22,23], and depressive symptoms [24,25,26,27]. Similarly, exposure to blueness (i.e., lakes, rivers, sea) has been linked to mental health and well-being [28,29,30,31,32,33,34].

Rare in this field are studies that examine the impact of “brownness” (i.e., elements of the desert environment) on health—despite the fact that these natural landscapes represent more than 35% of Earth’s land cover and are home to over 20% of the global population [35]. Work from evolutionary psychology and biophilic principles generally supports the idea that a negative association would exist, given the lack of cues of resource availability, refuge, or restoration that these environments contain [22,36]. Recent research in support of this negative association includes an experimental study that showed a depleting effect of observing images of desert landscapes on motivation to change bad habits [37]. Another experimental study showed that college students preferred deserts less and believed them to be less restorative than coniferous forest and tundra biomes [38]. However, to the best of our knowledge, the link between brownness and health has not been explicitly assessed with larger sample sizes of participants who actually live in and are familiar with deserts.

The dominant focus on greenness in the environmental psychology and epidemiology literature is understandable, but limited. Underlying theories, such as the savanna and forest hypotheses [39,40,41], posit an innate human preference for green environments, and are commonly called upon to explain the restorative and health-promoting effects of greenness [38]. These theories are less relevant to the study of nature contact and health in arid regions, where the attributes of nature, such as biodiversity and vegetation characteristics (e.g., tree height and structure), are vastly different from those in regions with temperate climates [42]. Moreover, land use and climate changes are projected to increase the global desert surface area to 48% by the end of the century, with 78% of the estimated change occurring in developing countries [43]. As the world urbanizes and climate change takes place, human contact with green natural environments is likely to become increasingly rare and socially structured. In order to better understand the ways in which expected changes to the global environmental landscape will affect human health, we must investigate the ways in which desert landscapes are associated with physical health outcomes, whether these associations come from the presence of these arid environments or the lack of exposure to green ones, and the ways in which even small amounts of green that exist within desert landscapes may still provide health benefits.

Here, we aim to answer these questions in the context of glucose dysregulation, a risk factor for type 2 diabetes. Type 2 diabetes contributes substantially to the global burden of disease [44] and is more prevalent among individuals in social disadvantage (e.g., racial or ethnic minorities, living in poverty) [45]. A growing body of evidence supports protective associations between greenness and diabetes prevalence [46,47,48,49], diabetes incidence [44,50,51], blood glucose [52], and glucose tolerance [48]. However, it is currently unknown whether there is an adverse association between brownness and fasting glucose, or whether these relationships persist with the small amounts of vegetation that occur in desert environments. We hypothesized that the presence of brownness, and not the absence of greenness, is associated with elevated (harmful) fasting glucose, and that the presence of greenness, even in the small amounts that occur in desert environments, is associated with lower (protective) levels of fasting glucose. We examined these hypotheses using data from a cross-sectional cohort of nursing students living in El Paso, Texas, which is located within the *Chihuahuan* desert in the United States southwest.

## 2. Methods

### 2.1. Study Population

We used baseline measurements from the Nurse Engagement and Wellness Study (NEWS), which has been described in detail elsewhere [53]. In short, the NEWS consists of a prospective cohort of 517 women and men between 18 and 55 years of age enrolled during the first semester of the Bachelor of Science in Nursing (BSN) program at the University of Texas at El Paso. Measurements included fasting glucose levels, body mass index, physical activity, as well as sleep duration and quality. Participant enrollment occurred between August 2015 and May 2018. Nursing students were recruited via emails, posters, flyers, media outlets and information sessions and provided informed consent prior to participation. This study was approved by institutional review boards at the University of Texas at El Paso (857149–1) and Harvard University (16–0080).

### 2.2. Study Assessments

*Fasting Glucose*: Glucose measurements were conducted after an overnight fast at the Biobehavioral Research Laboratory at the University of Texas at El Paso. A Cholestech LDX^®^ Analyzer (Cholestech Corporation, Hayward, CA, USA) was used to measure glucose levels on Lipid Panel plus Glucose Panel cassettes, across a range of 2.8–27.8 mmol/L, on approximately 35 μL of whole blood. The Cholestech LDX Analyzer measures glucose concentrations via an enzymatic method that uses glucose oxidase to catalyze the oxidation of glucose peroxide. Glucose measurements using this method have been found to be in good agreement (95% CI: 92–100%) with plasma glucose measurements conducted via a hexokinase method on a Hitachi 917 analyzer [54]. The instrument was calibrated immediately prior to each measurement following the protocol provided by the manufacturer.

*Greenness*: A proxy for chronic exposure to neighborhood-level greenness was calculated with a cumulative opportunity approach, using Euclidean buffers centered around participants’ residential addresses [55]. Greenness was defined as the normalized difference vegetative index (NDVI) in 30 m^2^ pixels using cloud-free Landsat 7 satellite imagery from a single summer day in 2016 [56]. Negative NDVI values were present in less than 5% of the study region. These were reclassified as missing data to avoid blue space (i.e., the Rio Grande river and irrigation canals) degrading greenness values and confounding results. Pixel values were averaged across buffer radii that corresponded to the amount of healthy and leafy green vegetation within a 3 min (250 m), 6 min (500 m), 120 min (1000 m) and 35 min (3000 m) walk for a healthy adult in their 30s [57]. Since there was insufficient evidence from previous research to define an optimal buffer size, we used multiple sizes and explore relationships across them.

**Figure 1:**
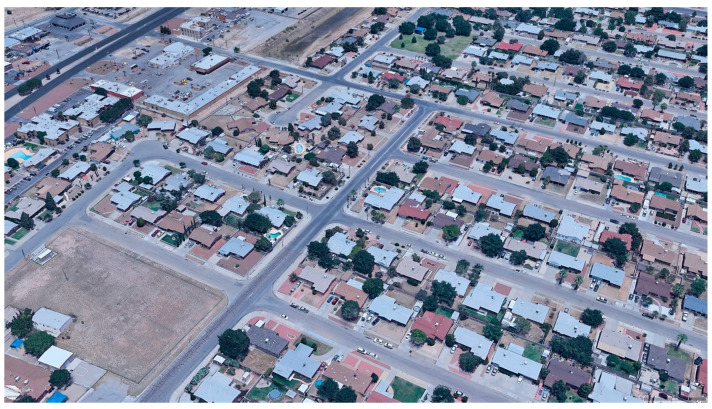
Aerial image showing examples of brownness, greenness, and grayness in a residential area of El Paso, Texas, in June, 2019. Image source: GoogleEarth^®^, © 2020 Google; © 2020 INEGI.

*Brownness*: Given the native desert landscape of El Paso, Texas, brownness was defined as the surface area that was not covered by vegetation or impervious surfaces (Figure 1). We calculated brownness in each 30 m^2^ pixel with the following equation:

Brownness=1.00− Greenness−Impervious

Here, greenness references NDVI values that range from 0 (no green) to 1 (maximum green). Impervious (“grayness”) represents the percentage of concrete, buildings, and natural rock (i.e., contiguous surface outcroppings) that do not allow water infiltration. Our operationalization of “brownness,” therefore, includes only soil, sand, and other natural non-living moveable materials. Impervious data were obtained from the National Land Cover Database (NLCD). These data were developed by the Multi-Resolution Land Characteristics (MRLC) Consortium (www.mrlc.gov) and displays 80% classification accuracy [58]. The pixel values were then averaged across the same buffer sizes that were used in the residential greenness estimations.

*Covariates*: An array of sociodemographic and health behavioral data were obtained through questionnaires with NEWS participants. Federal poverty status and the highest level of maternal education were used as indicators of socioeconomical status. Duration of physical activity by activity level (e.g., walking versus swimming or running), sleep activity by duration and subjective levels of quality were used to assess the role of health behaviors as potential intermediates of the association between greenness, brownness and fasting glucose [44]. Physical activity variables were used to estimate combined MET minutes per week [59].

### 2.3. Statistical Analyses

Primary analyses were restricted to participants under the age of 40, as individuals older than 40 years were considered more likely to have advanced states of illness, multiple comorbidities, and different behavioral patterns than the majority of the cohort [60]. The number of participants in the cohort that were 40 years or older was small (*n* = 19, 3.7% of the entire study). A further 28 participants were excluded for lack of outcome data, and 14 participants for lack of exposure data, or reporting primary residence addresses outside of the greater El Paso, Texas area. The final analytical sample with complete data was 456.

We assessed relationships between exposure to greenness, brownness, and grayness exposures within different buffer sizes (e.g., 250, 500, 1000 and 3000 m) and fasting glucose using linear regression models. Models included a natural cubic spline for age, indicator variables for gender and Hispanic ethnicity, categorical variables for race (categories for “White”, “Black”, “Asian”, “Native American or Alaskan Native”, and “Native Hawaiian or Other Pacific Islander”) and maternal education level (categories for “No-High School”, “Some High School”, “High School Graduate or GED”, “Some College or Technical School”, and “College Graduate or Higher”), and ratios of self-reported household income over the federal poverty threshold for individual socioeconomic status. These ratios were calculated by dividing the self-reported household income over the poverty thresholds as defined by the U.S. Census Bureau for 2016. This ratio is a function of household size and represents the relative position of an individual’s household income to the national poverty threshold and was entered in the models as a continuous variable. Greenness, brownness, or grayness values were entered in separate models as continuous variables, as well as in combinations of two of the three values. Given the relationship of the three values (summing to 1 or 100%), the parameters of models with a combination of two of these values are interpreted as the change in the outcome for an increase in exposure conditional on the other exposure in the model being constant, or in other words the exposure value left out of the model would have to decrease for any increase in the exposures in the models. For example, in the model including brownness and grayness, their respective model parameters are interpreted as the change in glucose for an incremental increase in brownness (or grayness) and corresponding incremental decrease in greenness, holding grayness (or brownness) constant. Potential non-linearities in exposure response were tested by fitting the exposure as categorical quartiles with the lowest quartile as the reference group in the same models as above and in a generalized additive framework using a penalized spline term.

In separate models, we added BMI, self-reported sleep quality, or physical activity levels to compare effect estimates with base models and determine potential mediation. We also assessed potential associations between exposure variables (i.e., greenness and brownness) and each of these potential mediating variables by treating each mediator as a dependent variable while adjusting for the same covariates as in the base model. Potential effect modification between exposure variables and glucose levels by physical activity and sleep quality was also assessed though interaction terms.

## 3. Results

The sample consisted of 456 participants who were younger than 40 years of age. The mean age was 24.2 years (SD = 4.5). A total of 80% were female and 91% identified as Hispanic or Latino (Table 1). A total of 85% of participants lived at their current address for two years or more and 66% for at least five years.

There were differences in the distribution of covariates across greenness quartiles. Significant differences were seen for age with younger participants tending to reside in greener areas (*F*(3, 453) = 3.21, *p* = 0.03) (Table 1). There was also a non-significant trend for BMI to be higher in greener quartiles. Similarly, we observed differences in the distribution of covariates across brownness quartiles (Supplementary Table S1). Participant’s maternal education tended to be higher in areas of lower brownness (*F*(3, 453) = 4.26, *p* = 0.005) and physical activity tended (non-significant) to be higher in higher brownness.

**Figure 2:**
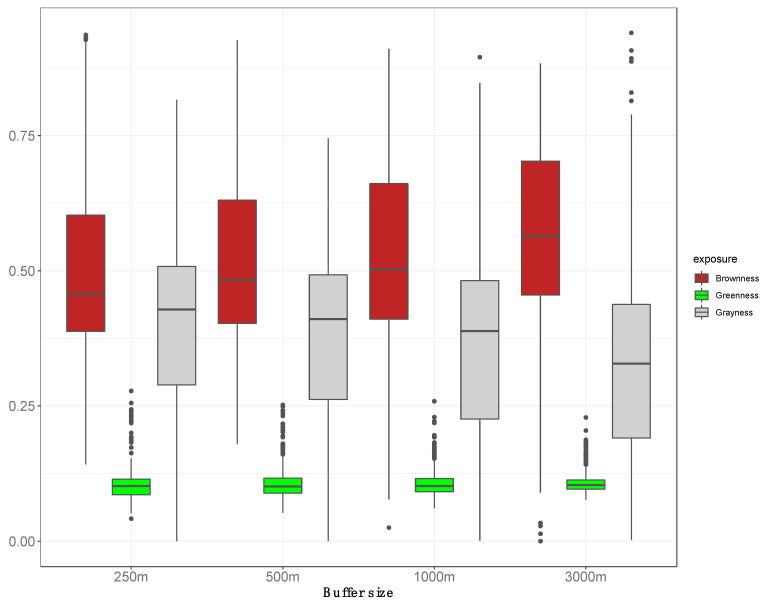
Boxplots for the distribution of brownness, greenness and grayness values summarized across different buffer sizes (e.g., 250, 500, 100, 3000 m) around participants homes. Greenness was measured with NDVI scores and brownness as the remaining surface area after subtracting the fractions of green surface and impervious (gray) surface from the total area of the buffer. The amount of impervious surface was obtained from the National Land Cover Database (NLCD) developed by the Multi-Resolution Land Characteristics (MRLC) Consortium (www.mrlc.gov).

Residential exposure data varied more for brownness than greenness across the sample (Figure 2). At the 250 m buffer, brownness values averaged near 0.51 (SD = 0.17) and ranged from 0.14 to 0.94. Greenness values at the 250 m buffer averaged near 0.11 (SD = 0.03) and ranged from 0.04 to 0.28. Grayness values averaged near 0.39 (SD = 0.17) and ranged from 0.00 to 0.82 at the same buffer size. Summary statistics for the three exposure values by buffer size are also available in Supplementary Table S2.

At the 250 m buffer, brownness was not associated with fasting glucose levels when it was modeled independently or in models adjusted for greenness (Table 2). However, when modeled together and in the absence of greenness, both brownness and grayness were positively associated with fasting glucose. An interquartile increase in brownness at the 250 m buffer size, holding grayness constant (i.e., same incremental decrease in greenness), was associated with a 6.6 mg/dL increase in fasting glucose levels (95% confidence interval (CI): −0.1, 13.6). Results at the 500 and 1000 m buffers were similar, but associations weakened at the 3000 m buffer size (Supplementary Table S3).

**Figure 3:**
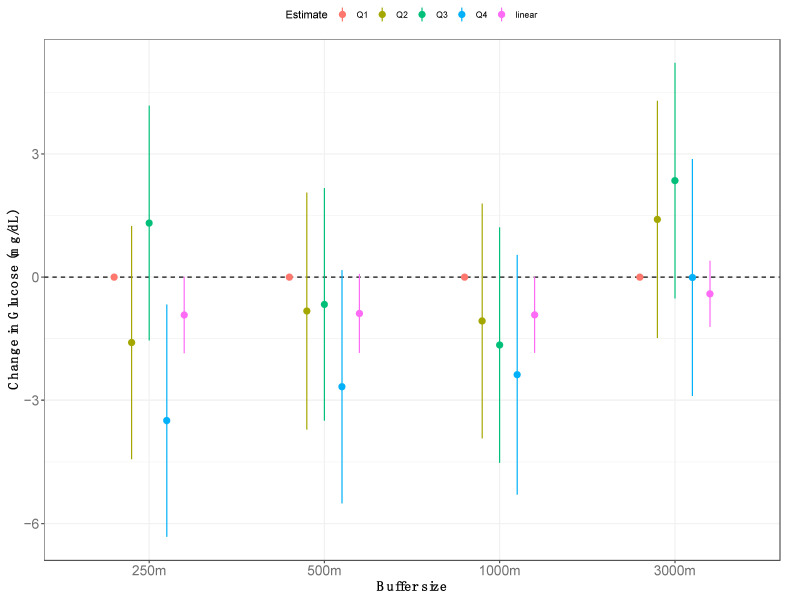
Estimated changes in glucose levels associated with exposure to greenness measured at different buffer sizes (e.g., 250, 500, 1000, and 3000 m) from linear regression models adjusted for age, gender, Hispanic ethnicity, race, and socioeconomic status (maternal education level and income over poverty ratio. Results are presented for quartiles of exposure with the first quartile of exposure as the referent category with dots representing the effect estimate and whiskers representing 95% confidence intervals. Results from a linear term for the exposure as a continuous variable (in pink) are also presented for change in glucose associated with an interquartile range increase in exposure at each buffer size.

**Figure 4:**
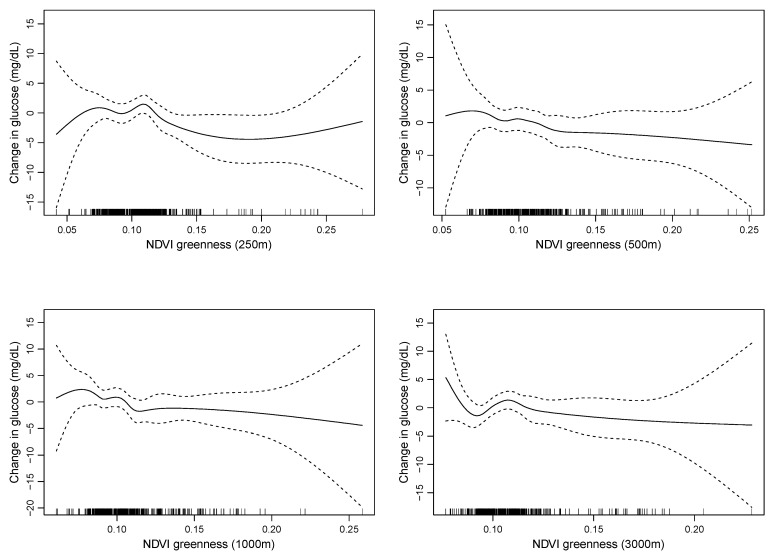
Penalized splines for the association between greenness measured at difference buffer sizes and changes in fasting glucose levels from generalized additive models adjusted for age, gender, Hispanic ethnicity, race, and socioeconomic status (maternal education level and income over poverty ratio). The solid line represents the change in glucose levels (*y* axis) as a function of the exposure (*x* axis) with tick marks on the *x* axis representing individual data points of the exposure distribution. Dotted lines represent 95% confidence intervals for the change in glucose levels.

A significant inverse association was observed between glucose levels and greenness quartiles at the 250 m buffer size with comparable effect estimates for the 500 and 1000 m buffers (Figure 3). The difference between the top and bottom quartiles at the 250 m buffer was associated with a 3.5 mg/dL decrease in glucose levels (95% confidence interval: −6.2, −0.8). Significant effect estimates for a linear greenness term were also seen at the 250 and 1000 m buffer sizes. The association between greenness and glucose was not affected by the inclusion of brownness or grayness (Table 2). The linearity of the relationship between fasting glucose and greenness varied by buffer size, with the relationship being closest to linear for the 500 m buffer (see Figure 4).

Associations between fasting glucose and BMI, sleep quality, sleep duration, and physical activity were observed in the expected directions, with lower BMI, higher physical activity and sleep duration and better sleep quality associated with decreased glucose levels. However, only the association with BMI was statistically significant (Supplementary Table S4). Associations of brownness and grayness with fasting glucose were unaffected by the inclusion of these factors into the models (Supplementary Table S5). The association between greenness and fasting glucose was also unaffected by the introduction of, sleep quality, sleep duration or physical activity into the model. The association was slightly stronger with the inclusion of BMI in the model, which was in line with the positive association observed between greenness and BMI (Table 3). Interaction terms between greenness and BMI, sleep quality or physical activity were not statistically significant (*p* > 0.05). Increase in greenness was associated with increased odds of better sleep quality, and increased odds of having physical activity levels greater or equal to 500 MET-minutes per week compared to less, but CIs for the associations included the null.

## 4. Discussion

In this study, we used cross-sectional data from healthy young adults living in El Paso, Texas to examine whether the presence of brownness, and not the absence of greenness, around the residences of participants is associated with elevated fasting glucose, and whether the presence of greenness is associated with lower fasting glucose within desert environments. We did not observe an association between brownness and fasting glucose when brownness was modeled independently or when greenness was included as a covariate in the model. However, when brownness and grayness were modeled together, we did find a positive association between brownness and fasting glucose. Given the fact that when brownness and grayness are modeled together, they vary at the expense of greenness, the positive association of fasting glucose with brownness was interpreted as the result of an absence of greenness, rather than an adverse association stemming from the presence of brownness (or grayness for that matter). We also observed an association between higher levels of greenness around participants’ homes and lower levels of fasting glucose after adjustment for potential confounders and mediators. This association was relatively consistent across 250, 500, and 1000 m buffer sizes, but weaker at 3000 m.

The primary contribution of these findings is preliminary epidemiological evidence linking greenness with lower levels of fasting glucose within a desert environment. In contrast to the vast majority of studies on greenness and health, we conducted our study in a desert, where average levels of greenness are very low and remaining land cover is mostly “brown” and “gray”. Specifically, our results show that in El Paso, Texas, where the median NDVI at 250 m was 0.10 (IQR, 0.03; min, 0.04; max, 0.28), a difference in NDVI between the top (NDVI > 0.11) and bottom (NDVI < 0.09) quartiles was associated with a 3.5 mg/dL reduction (95% CI: −6.2, −0.8) in fasting glucose. Surprisingly, the magnitude of this association with fasting glucose is consistent with two recent studies conducted in non-desert environments, where the range and average exposure to greenness were much greater than in our study [48,49]. In a prospective cohort of 6807 pregnant women in Wuhan, China, where the median NDVI at 300 m was 0.33 (IQR, 0.27; min, −0.09; max, 0.84), a difference in NDVI at 300 m between the top and bottom quartiles was associated with a 3 mg/dL reduction (95% CI: 3.8, 2.5) in fasting glucose, after adjusting for age, education, BMI, smoking, parity, season of conception, income, and urbanicity [48]. In a cohort study of 15,477 adults across 33 Chinese communities, where the median NDVI at 500 m was 0.29 (IQR, 0.17; min, 0.18; max, 0.82), a 0.1 increase in NDVI was associated with a 1.29% reduction (95% CI: 1.05, 1.55) in fasting glucose after adjusting for age, sex, education, income and ethnicity [49]. The fact that these associations between greenness and fasting glucose are similar—despite the fact that the local amounts of greenness in El Paso, Texas are smaller than the amounts in these locations—lends credence to the idea that these associations exist with respect to relative levels of greenness, rather than absolute levels.

Our results are also consistent with a broader set of studies that show that greenness closer to the home improves diabetes-related health outcomes. Pescador Jimenez et al. [61] observed that Massachusetts teenagers living in the highest quintile of residential greenness displayed lower insulin resistance than those in the lowest quintile. Similar to our findings, these differences were observed only for small 90 and 270 m radial buffers around the home. No associations were found for 1230 m buffers. Our results are also in line with other emerging work on the association of greenness and diabetes-related risk factors. Israeli children and teenagers who spent approximately two hours more per week in green space displayed a 0.5 mg/dL decrease in fasting glucose levels (95% CI: 0.9, 0.1) than other children, after adjusting for age, sex, birth weight, diet, parental risk factors, urbanity, and socioeconomic status [47]. Among teenagers in Germany, a 0.2 NDVI increase in a 1000 m buffer was associated with lower insulin resistance after adjusting for BMI, smoking, physical activity, and study area/individual characteristics.

Other epidemiological studies suggest that greenness suppresses a range of risk factors associated with type 2 diabetes, including poor sleep [9,12,46] and physical inactivity [62,63]. The results of our study among predominantly young Hispanics living in the desert were consistent with these findings in terms of direction of effect estimates, but we did not observe statistically significant associations between greenness and physical activity levels or sleep quality.

### 4.1. Implications for Theory

Several theories grounded in evolutionary psychology posit a human preference for natural environments [4,36,39,41,64,65] and are commonly called upon to explain the link between greenness and health [63,66,67]. Some of these theories, such as the savanna [41] and forest hypotheses [39], specifically propose a human preference for green environments, while others describe a preference for natural landscapes that offer conditions for survival [36], parasympathetic nervous system activation [4], or attention restoration [64]. Desert landscapes have not typically been included in the studies that use these hypotheses as their theoretical foundation. Brown environments do not fall into the categories of natural environments that are typically considered “beneficial” and full of available resources given our evolutionary past. Thus, to the best of our knowledge, the association of “brown” environments with health has not been tested using an epidemiological approach until now. When operating purely from the perspective of the biophilia hypothesis [65], a reasonable conclusion might be that desert environments represent harsh and inhospitable landscapes, and that proximity and exposure to them may therefore result in negative health outcomes. However, in our study, residential brownness was not associated with fasting glucose after adjusting for age, race, Hispanic ethnicity, socioeconomic status, and BMI.

One possible explanation for the lack of a significant, adverse association between brownness and fasting glucose may be due to the characteristics of the population of this study. The cohort consisted of long-term residents of the study region, individuals who may have developed familiarity and increased preference for desert landscapes. Attachment to desert landscapes, and the sense of place that these individuals might feel towards their local environments could potentially moderate what might otherwise be negative impacts [68,69], highlighting the importance of the lived experience, relative to the evolutionary perspective, in explaining the nature–human health relationship. On the other hand, the fact that we observed an association of greenness with lower fasting glucose in a predominately brown environment (despite adjustment for commonly proposed behavioral factors such as physical activity and sleep) supports prevalent evolutionary psychological theory, and not necessarily the influence of developed preferences or familiarity. Therefore, further research on the role of familiarity and sense of place in the nature–health connection is necessary to disentangle the roles of evolutionary psychology from those of the lived experience.

An unresolved question in the literature is the pathway connecting greenness and glucose/insulin-related mechanisms. Here, as in most of the literature on greenness, we rely on stress-centered phylogenetic perspectives to posit a pathway mediated by the activation of the hypothalamic–pituitary–adrenal (HPA) axis that leads to metabolic alterations through inflammatory processes [70]. However, it is also plausible that environmental cues of safety in green calm environments that in turn could signal cues of “readiness for regeneration” would also activate the hypothalamic–pituitary–gonadal (HPG) axis, which in turn affects metabolic processes through the regulation of sex hormones and other hormonal processes [71,72]. Future research should focus on the exploration of these alternative pathways connecting greenness and metabolic health. 

### 4.2. Implications for Practice

Our study suggests that in the desert, even small-scale greening interventions may provide a protective factor for health. The fact that the association between greenness and fasting glucose in El Paso, Texas is comparable to results from studies in China [48,49]—despite marked differences in the range of greenness levels—suggests that exposure levels to greenness should be considered relative to a local norm. It may be that the most relevant predictor for health is a change or difference in relative levels of nearby greenness, in contrast to a consideration of the absolute value of greenness without taking this context into account. In other words, greening interventions in deserts may be protective even if they are constrained to relatively small increases in vegetative cover, given the local context. The possibility that even small increases in greenness might improve health in deserts where levels of precipitation are low, and artificial irrigation is costly, supports the feasibility of policy-driven interventions in these environments.

In this regard, our results also have implications for vulnerable communities, which are often exposed to greater environmental injustices, including low levels and quality of greenness [73]. Desertification will increase as climate change continues, particularly in developing countries where vulnerable communities will be more likely to be exposed to brownness and limited amount of greenness [43]. Because populations of higher socioeconomic status will be more able to move or protect themselves from a changing climate, the “browning” of our globe may further exacerbate the poor living conditions of marginalized populations [42].

Our findings lend themselves to three practical conclusions that provide hope to public health officials battling these changes. First, we found associations between very small increases in greenness and reductions in fasting glucose, which is relevant to a disease that is rapidly rising in low-income populations [74]. Second, we found an association of greenness with lower fasting glucose specifically within desert environments. Third, our assessment was in a cohort of mostly Hispanic people who are at higher risk of diabetes [75] and are already concentrated in the desert regions of the U.S. [76].

### 4.3. Strengths and Limitations

The results of our study need to be interpreted in light of several limitations. The cross-sectional design of our study limited our ability to assess the assumed temporal order and directionality of the associations between exposure, potential mediators and the outcome of interest. The positive association between greenness levels and BMI, may have been the result of residual confounding. Our study may also have been underpowered to asses more modest associations between the exposure and potential mediators. The self-reported nature of data on potential mediators is also a limitation. Further, our sample of nursing students was dominated by females of a specific sociodemographic makeup that was higher and non-representative of the general population and therefore limits the generalizability of our results. Additionally, we assessed associations of greenness and brownness based on residential address information recorded at study enrollment days apart from the fasting glucose assessment. We did not have information on complete residential histories prior to study enrollment, which could have introduced a possibility for exposure misclassification. The interpretation of our results assuming that fasting glucose variation was associated with the long-term exposure to residential greenness/brownness, is subject to this potential exposure misclassification, though it is likely to be non-differential with respect to the outcome.

Our greenness measure leaves some room for improvement. Sensitivity analyses across several buffer sizes revealed that the linearity of the relationship between greenness and fasting glucose varied by the geographic scale in which greenness was measured. Consequently, we focused our analysis on greenness exposure measured in 250 and 500 m radial buffers, which exhibited the strongest linear relationship with fasting glucose but limited our direct comparison with published results from other studies with different buffer sizes. The use of NDVI as the metric for greenness has a series of limitations as well. NDVI does not allow for disentanglement of the type and quality of vegetation. Variations in NDVI in desserts have also been claimed to represent biological soil crusts as much as or more than actual leafy green vegetative cover [77]. Future work should consider alternative greenness measures, including Google Street View imagery or tree canopy cover. We were also not able to measure time spent at locations others than home, so our assessments were constrained to cumulative exposure calculations based upon residential location.

Our brownness measure was an early and imperfect attempt to measure the impacts of deserts on health. Values were calculated as the land cover not included in satellite measures of greenness (NDVI) or grayness (impervious surfaces). Some scenic attributes of deserts were excluded from these calculations, notably cohesive surface rock outcroppings. Further work with more comprehensive and sophisticated measures of brownness is necessary to rule out associations between brownness and fasting glucose.

We acknowledge that the differences in fasting glucose levels between greener and less green residential environments here and in other studies are rather modest. It is unclear whether associations across subgroups of individuals such as those who are physiologically compromised or socially vulnerable are present. Future research is needed to verify the true causal impact of greening on diabetes risk factors with more longitudinal or experimental study designs. Also needed are studies that determine the effect size of greenness exposure on glucose across different subgroups from the general population.

Despite these limitations, our study has several strengths. To the best of our knowledge, this is the first analysis of the association between residential brownness and health, and also the first examination of the ways in which small amounts of greenness are associated with diabetes risk factors in arid environments. Identifying an association between greenness and glucose levels at the lowest levels of greenspace exposure increases our confidence in a robust association between these variables.

## 5. Conclusions

In contrast to what the biophilia hypothesis and traditional theories from evolutionary psychology might predict, we did not observe a negative association of residential brownness (sand, soil) with fasting glucose in a cohort of young adults living in the desert region of El Paso, Texas. This may be due to the moderating influence of preference, familiarity, and sense of place that this population has for the desert environment in which they live. We did observe an association between residential greenness and fasting glucose. Adjustment for socioeconomic status, BMI, sleep quality, sleep duration, and physical activity did not affect this association. Despite the low levels of greenness in this desert environment, effect sizes were similar to those reported from regions with considerably higher levels of greenness. Our findings support the conclusion that the beneficial health associations of residential greenness should be considered in relative vs. absolute terms, and within the greater context in which this greenness exists. Even small absolute changes in greenness may have health implications within desert environments, given the fact that these differences could represent substantial relative changes in these areas. Further research is necessary to confirm that there is a protective effect of low levels of greenness on glucose and other diabetes-related pathways in desert environments. However, our findings support the feasibility of small-scale greening interventions, especially as climate change increases the likelihood that large, potentially vulnerable segments of the global population may live within desert environments, and experience greater daily exposure to desert landscapes.

## Figures and Tables

**Table 1:** Demographic characteristics of the Nurse Engagement and Wellness Study (NEWS) analytical sample in the current study (*n* = 456).

Characteristic	Overall	Greenness (Q1) **	Greenness (Q2) **	Greenness (Q3) **	Greenness (Q4) **	ANOVA F-Test *p*-Value
Age (years), mean ± SD	24.20 ± 4.53	25.08 ± 4.97	23.67 ± 4.10	24.54 ± 4.94	23.51 ± 3.88	0.03
BMI (kg/m^2^), mean ± SD	25.38 ± 5.36	25.01 ± 5.53	24.60 ± 4.74	26.33 ± 6.04	25.58 ± 4.92	0.08
Male, *n* (%)	92 (20.2)	24 (21.1)	20 (17.5)	28 (24.6)	20 (17.5)	0.50
Race, *n* (%)						0.17
American Indian or Alaska Native	10 (2.2)	4 (3.5)	1 (0.9)	2 (1.8)	3 (2.6)	
Asian	9 (2.0)	3 (2.6)	2 (1.8)	2 (1.8)	2 (1.8)	
Black or African American	11 (2.4)	6 (5.3)	4 (3.5)	0 (0)	1 (0.9)	
Native Hawaiian or Other Pacific Islander	1 (0.2)	1 (0.9)	0 (0)	0 (0)	0 (0)	
White	425 (93.2)	100 (87.7)	107 (93.9)	110 (96.5)	108 (94.7)	
Hispanic or Latino, *n* (%)	413 (90.6)	98 (86.0)	105 (92.1)	104 (91.2)	106 (93.0)	0.27
Maternal Education, *n* (%)						0.08
No High School	49 (10.7)	19 (16.7)	9 (7.8)	7 (6.1)	14 (12.2)	
Some High School	35 (7.7)	8 (7.0)	3 (2.6)	11 (9.6)	13 (11.4)	
High School Graduate	97 (21.0)	19 (16.7)	22 (19.3)	26 (22.8)	30 (26.3)	
Some College	129 (28.2)	29 (25.4)	41 (36.0)	36 (31.6)	23 (20.2)	
College graduate	142 (31.1)	38 (33.3)	38 (33.3)	32 (28.1)	34 (29.8)	
Income/Poverty *, mean ± SD	2.27 ± 1.83	2.26 ± 1.94	2.11 ± 1.64	2.43 ± 1.91	2.30 ± 1.84	0.93
Physical activity * (MET hours/week), mean ± SD	63.6 ± 73.9	59.2 ± 57.9	59.3 ± 73.4	69.0 ± 91.3	67.7 ± 69.9	0.26
Sleep (hours) *, mean ± SD	6.39 ± 1.08	6.42 ± 1.06	6.32 ± 1.09	6.37 ± 1.12	6.46 ± 1.07	0.70

* Information on income over poverty ratio, physical activity, and sleep duration was missing for 67 (14.6%), 6 (1.3%) and 46 (10.1%) participants, respectively. ** Quartiles are indicted by Q1, Q2, Q3, and Q4.

**Table 2:** Associations between fasting glucose levels and greenness, brownness and impervious surface area. Results shown are from linear regression models using the 250 m buffer and that included each exposure measure separately, and models that included combinations of two of the exposure measures.

Model	Exposure(s)			Change in Fasting Glucose, mg/dL (95% CI)		
				Brownness	Greenness	Grayness
1	●			−0.01 (−0.07, 0.05)		
2	●	◆		−0.01 (−0.07, 0.05)	−0.32 (−0.63, −0.01)	
3	●		■	0.31 (−0.02, 0.63)		0.32 (0.01, 0.63)
4	◆				−0.32 (−0.63, −0.01)	
5	◆		■		−0.31 (−0.63, 0.02)	0.01 (−0.05, 0.07)
6			■			0.02 (−0.04, 0.08)

◆, greenness; ●, brownness; ■, grayness; all models adjusted for age, gender, race/ethnicity, ratio of household income over poverty and maternal education. Effect estimates for linear terms are for a 0.01 (1%) increase in exposure values.

**Table 3:** Associations between greenness measured at the 250 m buffer size, and potential mediators for the association with fasting glucose. Results presented from models where each mediator was modelled as the outcome of interest, both as continuous and binary* variables in linear and logistic models, respectively.

Potential Mediator	Association with An IQR Increase in Greenness	
	Change (95% CI)	OR (95% CI)
BMI (kg/m^3^)	0.49 (0.00, 0.97)	1.21 (0.96, 1.52)
Physical Activity (percent change) **	5.1 (−5.5, 16.8)	1.42 (0.92, 2.19)
Sleep Duration (hours)	−0.04 (−0.14, 0.05)	
Sleep Quality		1.16 (0.95, 1.40)

* Binary variables defined as BMI>= and <30 kg/m^3^ physical activity as >= and <500 MET mins/week, and sleep quality (self-reported ‘strongly agree’ or ‘agree’ with good quality of sleep compared to ‘neutral’, ‘disagree’ or ‘strongly disagree’). ** Physical activity in MET mins/week was log transformed and results from the linear model for this outcome are presented as percent change.

## Data Availability

The data presented in this study are available on request from the corresponding author.

## Supplementary Materials

The following are available online at https://www.mdpi.com/1660-4601/18/2/520/s1, Table S1: Participant characteristics by quartiles of brownness within 250 m of their primary residence. Table S2: Summary Statistics for greenness, brownness and grayness values within 250 m, 500 m, 1000 m and 3000 m of primary residence. Values presented as percentages of total with the sum of greenness, brownness and grayness totaling 100% for each individual. Table S3: Associations between glucose levels and greenness, brownness and impervious surface area. Results shown at the 500 m, 1000 m and 3000 m buffers, from models^*^ including exposure measures separately, and models with each combination of two of the exposure measures. Table S4: Associations between fasting glucose levels and potential intermediates. Results shown with greenness measured at the 250 m buffer size included in the model. Table S5: Associations between fasting glucose levels and greenness, brownness and grayness at the 250m buffer size with additional covariates in the model. Effects presented per 0.01 (1%) increase in exposure values.
