# Supplementary material for: Associations of Residential Brownness and Greenness with Fasting Glucose in Young Healthy Adults Living in the Desert
Source: Int J Environ Res Public Health. 2021 Jan 10;18(2):520. doi: 10.3390/ijerph18020520 (PMC7826883; doi:10.3390/ijerph18020520)
Supplement: Supplementary file 1 [file ijerph-18-00520-s001.pdf]

Supplemental Materials

Table S1: Participant characteristics by quartiles of brownness within 250m of their primary residence.

Characteristic	Brownness Q1	Brownness Q2	Brownness Q3	Brownness Q4	ANOVA F-test p-value
Participants, n	114	114	114	114	
Age (years), mean±SD	23.82±4.18	23.54±3.84	24.82±5.21	24.62±4.70	0.10
BMI (kg/m^2), mean±SD	25.90±5.93	25.06±5.59	25.18±4.97	25.38±4.91	0.65
Male, n (%)	23 (20.2)	25 (21.9)	22 (19.3)	22 (19.3)	0.96
Race, n (%)					0.35
American Indian or Alaska Native	1 (0.9)	4 (3.5)	1 (0.9)	4 (3.5)	
Asian	0 (0)	5 (4.4)	3 (2.6)	1 (0.9)	
Black or African American	4 (3.5)	1 (0.9)	3 (2.6)	3 (2.6)	
Native Hawaiian or Other Pacific Islander	1 (0.9)	0 (0)	0 (0)	0 (0)	
White	108 (94.7)	104 (93.9)	107 (91.2)	106 (94.7)	
Hispanic or Latino, n (%)	103 (90.4)	106 (93.0)	99 (86.8)	105 (92.1)	0.40
Maternal Education, n (%)					0.005
No High School	11 (9.6)	7 (6.1)	12 (10.5)	19 (16.7)	
Some High School	5 (4.4)	9 (7.9)	6 (5.3)	15 (13.2)	
High School Graduate	26 (22.8)	20 (17.5)	25 (21.9)	25 (21.9)	
Some College	36 (31.6)	41 (36.0)	27 (23.7)	25 (21.9)	
College graduate	35 (30.7)	37 (32.5)	42 (36.9)	28 (24.6)	
Income/Poverty*, mean±SD	2.26±1.94	2.11±1.65	2.43±1.91	2.30±1.84	0.67
Physical activity* (MET hours/week), mean±SD	54.43±57.86	54.45±49.38	73.30±93.12	72.95±84.92	0.07
Sleep (hours)*, mean±SD	6.38±1.08	6.51±1.06	6.24±0.99	6.41±1.20	0.74

*Information on income over poverty ratio, physical activity, and sleep duration was missing for 67(14.6%), 6(1.3%) and 46(10.1%) participants respectively.

Table S2: Summary Statistics for greenness, brownness and grayness values within 250m, 500m, 1000m and 3000m of primary residence. Values presented as percentages of total with the sum of greenness, brownness and grayness totaling 100% for each individual.

Buffer Size	Greenness		Brownness		Grayness	
	mean±SD	median (IQR)	mean±SD	median (IQR)	mean±SD	median (IQR)
250m	10.62±3.17	10.22 (8.58, 11.48)	50.86±17.13	45.73 (38.80, 60.30)	38.53±17.23	42.83 (28.88, 50.81)
500m	10.73±2.93	10.13 (8.88, 11.67)	52.39±16.76	48.27 (40.26, 63.06)	36.87±17.58	41.01 (26.20, 49.24)
1000m	10.82±2.66	10.19 (9.16, 11.60)	53.63±17.00	50.30 (41.05, 66.13)	35.54±17.71	38.84 (22.58, 48.17)
3000m	10.90±2.10	10.40 (9.62, 11.32)	56.01±18.23	56.42 (45.53, 70.29)	33.09±18.70	32.82 (19.07, 43.78)

Table S3: Associations between glucose levels and greenness, brownness and impervious surface area. Results shown at the 500m, 1000mand 3000m buffers, from models* including exposure measures separately, and models* with each combination of two of the exposure measures.

Exposure variable	Change in Glucose, mg/dL (95% CI)		
	Greenness	Brownness	Grayness
		500m buffer	
Individual models	-0.32 (-0.65, 0.02)	-0.02 (-0.08, 0.04)	0.02 (-0.04, 0.08)
Greenness + brownness	-0.31 (-0.65, 0.02)	-0.01 (-0.07, 0.05)	.
Greenness + Impervious	-0.30 (-0.65, 0.04)	.	0.01 (-0.05, 0.07)
Brownness + Impervious	.	0.30 (-0.05, 0.65)	0.32 (-0.03, 0.65)
		1000m buffer	
Individual models	-0.38 (-0.75, -0.01)	-0.03 (-0.09, 0.03)	0.03 (-0.02, 0.09)
Greenness + brownness	-0.36 (-0.74, 0.01)	-0.02 (-0.08, 0.04)	.
Greenness + Impervious	-0.34 (-0.73, 0.04)	.	0.02 (-0.04, 0.08)
Brownness + Impervious	.	0.34 (-0.04, 0.73)	0.36 (-0.01, 0.74)
		3000m buffer	
Individual models	-0.23 (-0.70, 0.22)	-0.02 (-0.07, 0.04)	0.02 (-0.03, 0.07)
Greenness + brownness	-0.22 (-0.69, 0.25)	-0.01 (-0.07, 0.04)	.
Greenness + Impervious	-0.21 (-0.69, 0.28)	.	0.01 (-0.04, 0.07)
Brownness + Impervious	.	0.21 (-0.28, 0.69)	0.22 (-0.25, 0.69)

*All models adjusted for age, gender, race/ethnicity, ratio of household income over poverty and maternal education. Effect estimates for linear terms are for a 0.01 (1%) increase

Table S4: Associations between fasting glucose levels and potential intermediates. Results shown with greenness measured at the 250m buffer size included in the model.

Potential mediator	Change in glucose in mg/dL (95% CI)
BMI (per kg/m^3)	0.35 (0.16, 0.54)
Physical activity	
Binary variable*	-0.59 (-3.86, 2.68)
Continuous (per 100 MET mins/week increase)	-0.02 (-0.04, 0.01)
Sleep quality*	-1.80 (-3.81, 0.21)
Sleep duration (per hour increase)	-0.20 (-1.14, 0.74)

*Binary variables defined as physical activity as >= and <500 MET mins/week, and sleep quality (self-reported 'strongly agree' or 'agree' with good quality of sleep compared to 'neutral', 'disagree' or 'strongly disagree').

Table S5: Associations between fasting glucose levels and greenness, brownness and grayness at the 250m buffer size with additional covariates in the model. Effects presented per 0.01 (1%) increase in exposure values.

Additional covariate	Change in glucose in mg/dL (95% CI)		
	Brownness	Greenness	Grayness
Base model	-0.01 (-0.07, 0.05)	-0.32 (-0.63, -0.01)	0.02 (-0.04, 0.08)
+ BMI (per kg/m^3)	-0.01 (-0.07, 0.05)	-0.38 (-0.69, -0.07)	0.02 (-0.03, 0.08)
+ Physical activity			
As binary variable*	-0.01 (-0.07, 0.05)	-0.31 (-0.63, 0.00)	0.02 (-0.03, 0.08)
As continuous (MET minutes/week)	-0.01 (-0.07, 0.05)	-0.32 (-0.63, -0.01)	0.02 (-0.04, 0.08)
+ Sleep quality*	-0.01 (-0.07, 0.05)	-0.31 (-0.62, 0.00)	0.02 (-0.03, 0.08)
+Sleep duration (per hour increase)	-0.00 (-0.06, 0.06)	-0.29 (-0.64, 0.05)	0.01 (-0.05, 0.07)

*Binary variables defined as physical activity as >= and <500 MET mins/week, and sleep quality (self-reported 'strongly agree' or 'agree' with good quality of sleep compared to 'neutral', 'disagree' or 'strongly disagree').
